# Lumping and Splitting of Distribution Models Across a Biogeographic Divide Informs the Conservation of an Imperiled Fluvial Fish

**DOI:** 10.1002/ece3.71315

**Published:** 2025-04-25

**Authors:** Briant D. Nguyen, Jenna Messick, Anthony W. Rodger, Victoria Jackson, Christopher Butler, Andrew T. Taylor

**Affiliations:** ^1^ Department of Biology University of Central Oklahoma Edmond Oklahoma USA; ^2^ Oklahoma Department of Wildlife Conservation, Stream Program Porter Oklahoma USA; ^3^ Department of Biology Texas A&M University College Station Texas USA; ^4^ Department of Biology University of North Georgia Dahlonega Georgia USA

**Keywords:** biodiversity conservation, ecological niche modeling, ecological species concept, freshwater ecology, species distribution modeling, threatened species

## Abstract

Freshwater fishes are among the most threatened taxa in the world. A major challenge for the conservation and management of threatened fishes is scarce information regarding life history, habitat requirements, and the drivers of declines. Species distribution models (SDMs) that leverage existing occurrence records and geospatial data have aided in addressing these challenges. We used SDMs to better understand large‐scale distributional patterns of Bluntface Shiner ( *Cyprinella camura* ; BFS), a minnow facing declines across its range. We modeled the potential distribution of BFS based on natural, abiotic factors and existing occurrence records to identify landscape‐scale factors underlying their distribution. We also compared environmental conditions between their disjunct ranges east and west of the Mississippi River and examined model transferability when projecting models into opposing ranges. Our analyses revealed a naturally fragmented distribution both east and west of the Mississippi River, but populations to the east of the Mississippi River occupy streams with broadscale environmental conditions that differ from those to the west. Models projected across the Mississippi River did not reflect the contemporary range of BFS, underscoring differences in occupied niches on either side of the biogeographic divide and emphasizing the need for caution when projecting SDMs to novel ranges. Our results provide a baseline to gauge range loss of BFS, highlight areas of high suitability for conservation, and identify locations where further sampling or research may be warranted.

## Graphical abstract

The Bluntface Shiner ( *Cyprinella camura* ) lives in freshwater streams on either side of the lower Mississippi River, United States, which represents a large biogeographic divide for freshwater fishes. We used distribution modeling and a “lumping and splitting” approach to better understand the distribution, ecology, and conservation needs of this imperiled and data‐deficient species.

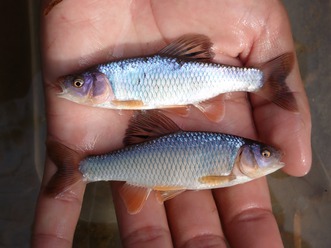

## 1 Introduction

Globally, freshwaters are remarkably biodiverse ecosystems with over 12,000 fish species existing in lakes and rivers, despite these waters making up < 0.01% of the Earth's water supply (Dudgeon et al. 2006; Nelson et al. 2016). In North America, there are over 1050 freshwater fishes, and riverine ecosystems are hotspots for aquatic biodiversity (Lundberg et al. 2000; Master et al. 1998). However, the extinction rate of freshwater fishes has increased eightfold in recent decades, and nearly 46% of North American species are at risk of going extinct (Burkhead 2012; Dudgeon et al. 2006; Jelks et al. 2008; Walsh et al. 2009). In light of unprecedented extinction rates, these animals require concerted research and conservation efforts to maintain global biodiversity and associated economic and social values (Walsh et al. 2009).

Habitat alteration is a major threat to river ecosystems that results in changes to natural stream hydrology, physiology, and water quality (Allan 2004; Dudgeon et al. 2006; Paul and Meyer 2001; Walsh et al. 2005). Streams are especially vulnerable given their connections to the surrounding landscapes (Hynes 1975). However, because natural processes of rivers are a function of the longitudinal (i.e., upstream to downstream), lateral (i.e., the floodplain and riparian zone), vertical (i.e., groundwater), and temporal dimensions (Ward 1989), there is inherent difficulty in identifying causal relationships between the landscape, riverscape, and what factors are driving losses of biota. In the last two decades, advancements in geographic information systems (GIS), availability of environmental datasets, and application of landscape ecology to river ecosystems have helped address these challenges (Brenden et al. 2006; Wang et al. 2006).

Species distribution models (SDMs; also known as ecological niche models [ENMs]) are valuable tools for understanding the species–habitat relationships that underlie species distributions. With SDMs, the fundamental niche of a species can be modeled by relating species occurrence records in the form of presence‐only, presence–absence, or pseudo‐absence to environmental predictors of the background area to estimate habitat suitability across a study area (Franklin 2010; Guisan and Thuiller 2005). Presence‐only models use species occurrences commonly curated by natural history museums or online species occurrence databases wherein corresponding data on species absence is unavailable (Elith et al. 2011; Pearson 2007; Phillips et al. 2006). A widely used SDM method implemented in Maxent (Maximum entropy method; Phillips et al. 2017) is advantageous for analyses using presence‐only data because it allows for tuning model inputs and settings to alleviate bias and optimize predictive performance (Kramer‐Schadt et al. 2013; Merow et al. 2013; Phillips et al. 2009). Maxent has been adapted for use in aquatic settings to identify important environmental associations of a species, elucidate the potential drivers of their decline, and provide a basis to assess the extent of decline (Allen et al. 2022; Bouska et al. 2015; Huang and Frimpong 2015; Key et al. 2021; Labay et al. 2011; Laman et al. 2018; McGarvey et al. 2021; Pont et al. 2015; Taylor et al. 2017). Distribution models can also be projected into novel spatial and temporal extents to provide inferences on the effects of environmental change or invasion into novel geographic regions (Bartnicki et al. 2021; Huang et al. 2016; Sundblad et al. 2009).

Species distribution modeling of data‐deficient stream‐dwelling fishes, such as the Bluntface Shiner (BFS; *Cyprinella camura* ), can provide important insights into species ecology and conservation status. BFS are native to two disjunct regions east and west of the lower Mississippi River, which serves as a biogeographic faunal divide for headwater stream fishes (Egge and Hagbo 2015). Early investigators of BFS taxonomy identified some apparent morphological differences between east and west populations, though genetic tests conducted at that time did not support delimiting these populations into subspecies or separate species (Gibbs Jr. 1961; LeDuc 1984). More recently, the mitochondrial gene cytochrome *b* (*cyt b*) was used to investigate the phylogeography of BFS, and populations from either side of the Mississippi River were not monophyletic. Interestingly, this *cyt b* phylogeny recovered two distinct lineages of BFS, with those to the east of the Mississippi River sharing a more recent common ancestor with Whitetail Shiner ( *Cyprinella galactura* ). These results raise the possibility of cryptic species‐level diversity within BFS (Egge and Hagbo 2015). Information gleaned from SDMs, such as differences in underlying ecological niches across the Mississippi River, could prove useful in supporting future taxonomic revision.

Although BFS populations occupy distant and disjunct areas, there are broad ecological similarities reported for BFS, with some nuanced habitat differences also noted. In both ranges, BFS are often associated with medium‐ to large‐sized streams with riffles and runs (Cross 1954; Etnier and Starnes 1993; Farr 1996; Wilkinson and Edds 2001). To the east, within the lower Mississippi River Basin, BFS occupy upland, headwater tributaries with swift flowing water over sand, mud, and gravel substrates (Farr 1996; Johnston 1999; LeDuc 1984; Mayden 1989; Ross and Brenneman 2001). To the west, within the Arkansas River Basin, BFS occupy high gradient streams with flowing waters over gravel and rubble substrates (Fuselier and Edds 1996; Metcalf 1989) but are less abundant in lowland streams with sand and mud substrates (Cross and Cavin 1971; Metcalf 1989; Wilkinson and Edds 2001). Across the entirety of their range, BFS spawning occurs during spring and summer months (Distler et al. 2014; Etnier and Starnes 1993; Miller and Robinson 2004; Robinson and Buchanan 2020; Ross and Brenneman 2001). As crevice spawners, BFS require streams possessing larger substrates (i.e., gravel) for successful reproduction (Johnston 1999; Mayden 1989). Many BFS occurrence records are within larger mainstem rivers with nearby access to smaller tributary streams. In the Arkansas River Basin where drought conditions are common (Matthews 1988), access to larger sized streams may be important for BFS survival and reproduction.

Local declines of Bluntface Shiner have been documented (e.g., Cross and Braasch 1968), and populations in several states appear to be declining. For example, in Louisiana, BFS have not been captured in recent years (Robby Maxwell, Louisiana Department of Natural Resources, Pers. Comm.). In Oklahoma, the majority of historic BFS records exist in tributaries of the lower Arkansas River; however, field surveys in this area in 2022 yielded only two BFS detections (Anthony Rodger, Oklahoma Department of Wildlife Conservation, Pers. Comm.). A number of streams that historically held BFS in Missouri and Oklahoma flow into the state of Arkansas, yet no BFS have been documented in Arkansas since the late 1960s (Robinson and Buchanan 2020). As such, BFS are considered at risk of extirpation in Oklahoma, Missouri, and Louisiana (Louisiana Wildlife and Fisheries 2015; Missouri Department of Conservation 2015; Oklahoma Department of Wildlife 2015) and extirpated from Arkansas (Arkansas Game and Fish Commission 2015). Conversely, populations appear stable and secure in Kansas and Mississippi. According to the International Union for Conservation of Nature's global population assessment, BFS are of Least Concern, but the population trend is unknown. Given the ostensibly limited range and disjunct populations, any further declines are of great concern.

In this investigation, we estimated the potential distribution of BFS using Maxent and accessory packages within the R programming environment (R Core Team 2021) using a comparative “lumping” versus “splitting” approach to account for potential evolutionary and ecological diversity within BFS. Our first objective was to construct models of BFS's potential distribution and visualize them with suitability maps. We modeled each disjunct range independently (i.e., “splitting”) because of potential differences in ecology on either side of the lower Mississippi River and then modeled across the entire combined range to better encompass the entirety of conditions BFS may inhabit (i.e., “lumping”). Our next objective was to examine congruence and visualize differences in estimated distributions by projecting models built on either side of the lower Mississippi River into opposing areas. Our final objective was to compare the environmental conditions between the disjunct BFS ranges to better understand the ecological underpinnings of our model results. This study can help fill knowledge gaps related to BFS's environmental requirements, inform future conservation action by illuminating areas of high habitat suitability and provide a reference for assessing range loss.

## 2 Materials and Methods

For the modeling efforts detailed below, we compiled an ODMAP (Overview, Data, Model, Assessment, and Prediction; Zurell et al. 2020) protocol of our workflow as a supplement to the methods reported herein to ensure reproducibility (Supporting Information).

### 2.1 Study Area

**FIGURE 1:**
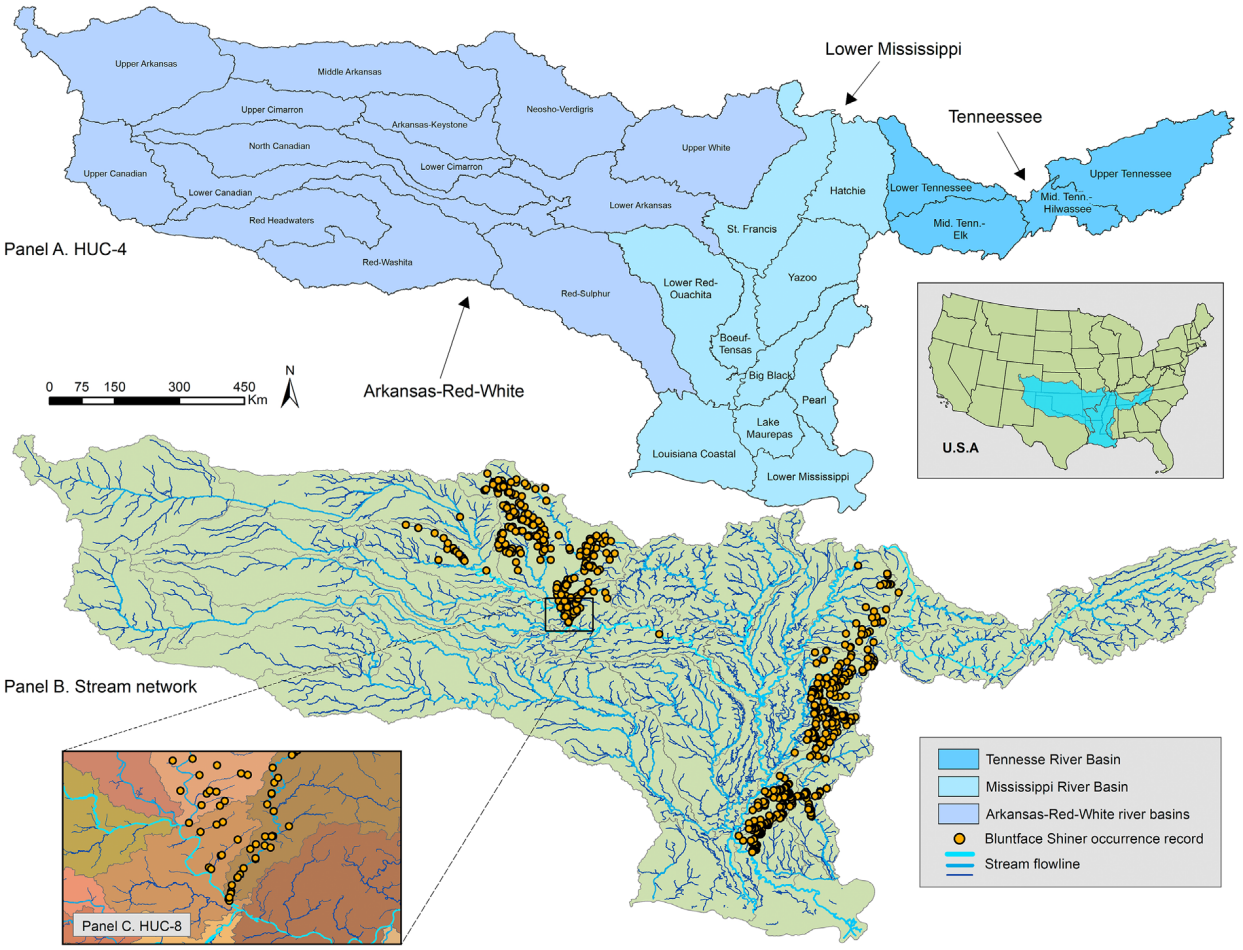
Study area encompassing watersheds of the Arkansas‐Red‐White, the Lower Mississippi, and the Tennessee river basins (Panel A). The distribution models were produced with streamflow lines (stream orders 1–4 not shown here) and occurrence records (Panel B). The occurrence and background records were divided by HUC‐8 for k‐fold cross‐validation (Panel C).

Our study area was determined by BFS occurrence records and the watersheds they occur within. To model within riverscapes, we used watershed boundaries delineated by the United States Geological Survey's Hydrologic Units (HUCs). HUCs are numerically coded by two‐digit to 12‐digit hydrologic unit codes (HUCs; the larger the HUC, the finer the watershed subdivision). We defined our study area extent to three two‐digit HUC regions that contained BFS occurrence records: the Arkansas‐Red‐White (11), Lower Mississippi (08), and Tennessee (06; Figure 1A). We created a model that treated the disjunct BFS populations as one (i.e., “lumping”) with these three HUCs joined together (denoted as ALL range). We also modeled the Arkansas‐Red‐White (ARW, to the west of the Mississippi River) and the Lower Mississippi and Tennessee (LMT, to the east of the Mississippi River) ranges separately (i.e., “splitting”). The study grain was individual stream segments within the National Hydrography Dataset Plus, version 2 (NHDPlusV2; McKay et al. 2012).

#### 2.1.1 Arkansas‐Red‐White (ARW)

The ARW spans from the Great Continental Divide to the Mississippi River across eight states (Colorado, New Mexico, Kansas, Oklahoma, Texas, Arkansas, Missouri, and Louisiana). The mountainous western portion of the ARW reaches elevations up to 4000 m, and the streams flow over igneous and metamorphic geology (Cain 1988; Kilsby et al. 1999). Within the central ARW are relatively low‐elevation prairie streams with bedrock and sedimentary rock geology (Cain 1988). Nearer to the Mississippi River Basin are the Ozark Plateau and Ouachita Mountains, which have rolling hills and mountains that reach elevations from 150 to 800 m with limestone, sandstone, and sedimentary substrates (Kilsby et al. 1999; United States Army Core of Engineers 1990). In the mountainous western extremes of the ARW, hydrology is driven in large part by snowmelt runoff during spring thaw, whereas in the prairie and Ozark regions, stream hydrology is primarily driven by rainstorm runoff and groundwater springs (Hays et al. 2016; Kilsby et al. 1999; Leasure et al. 2016; United States Army Core of Engineers 1990). Within these areas, intermittent and ephemeral streams are common (Matthews 1988).

#### 2.1.2 Lower Mississippi and Tennessee (LMT)

The LMT spans from the Appalachian Mountains to the Gulf of Mexico across 10 states (Virginia, North Carolina, Kentucky, Tennessee, Georgia, Alabama, Missouri, Arkansas, Mississippi, and Louisiana). The Lower Mississippi River Basin consists of meandering rivers abundant with tributaries, oxbows, and backwaters that ebb and flow across the Alluvial Plain from the confluence of the Ohio River toward the Gulf coast. Elevation ranges from sea level to 200 m with uplands that surround the fluvial valley (Etnier and Starnes 1993; Rittenour et al. 2007). The streams flow over fine sediments of sand, clay, silt, and some gravel deposits. The hydrology is driven by precipitation (Agriculture Research Service 2012; Fisk 1951) and flooding is common (Etnier and Starnes 1993). The Tennessee River Basin is geologically complex with highlands, plateaus, and mountainous expanses across the range. To the east, the Blue Ridge province reaches elevations > 1800 m, while to the west, the Highland Region averages around 300 m elevation (Etnier and Starnes 1993). In the low‐ to moderate‐gradient streams, sand, gravel, and bedrock substrates of limestone, chert, sandstone, and shale predominate, whereas the high‐gradient mountain streams flow over bedrock and boulder substrates with some sand and gravel deposits (Etnier and Starnes 1993; Rodgers 1953). Winter snowfall and summer rain provide ample precipitation throughout the year (Federal Energy Resources Commission 1981).

### 2.2 Occurrence Records

To collect existing BFS occurrence records, we queried online databases GBIF (https://www.gbif.org), Fishnet2 (http://www.fishnet2.net), IDigBio (https://www.idigbio.org), iNaturalist (https://www.inaturalist.org), and BISON (https://bison.usgs.gov). Given a marked paucity of public records for BFS in Oklahoma, we also requested records from state agencies (Oklahoma Department of Wildlife Conservation and Oklahoma Conservation Commission) and natural history museums (University of Oklahoma Sam Noble Museum and Oklahoma State University). We cleaned the combined dataset by removing replicates (199 records) and records that lacked both assigned coordinates and locality descriptions (30 records). We removed records with an invalid locality (i.e., those that had nebulous descriptors) and replicate georeferenced sites (54 records). We used GEOLocate v3.22 (Rios and Bart 2010) for 126 records that contained locality descriptions but no geographic coordinates. Using ArcMap v10.8.2 (ESRI 2022), we spatially joined all occurrence records to their nearest NHDPlusV2 stream segments and manually checked that occurrence records were linked to the proper stream. Erroneously joined records were identifiable by larger join distances or by mismatches with the record's locality information. We rectified these mistakes by visually inspecting the map for nearby streams and manually joining the occurrence record to the nearest stream segment that matched the locality information. In total, 762 BFS occurrence records remained for model analysis (Figure 1B).

### 2.3 Environmental Variables

We gathered environmental predictors from the datasets NHDPlusV2, StreamCat (Hill et al. 2016), and the Stream Classification System (SCS; McManamay and DeRolph 2019) to inform our distribution models. NHDPlusV2 is a constructed network of stream segments that span confluence‐to‐confluence and contains environmental information for streams of the conterminous United States at a 1:100,000 scale. StreamCat and the SCS were built upon the NHDPlusv2 stream network and provide a suite of additional environmental variables for each stream segment. Each dataset uses the same common identifier (COMID) to link their stored environmental information to stream segments. Environmental variables within these datasets vary in spatial scale ranging from stream segment level (a single streamline of the stream network; smallest spatial scale), catchment level (all surface area that drains to a single stream segment, excluding any upstream contributions; medium spatial scale), and watershed level (all surface area of connected catchments, including upstream contributions, that drain to a single stream segment; largest spatial scale). We selected variables spanning these different scales to capture local environmental conditions as well as broader, cumulative processes that typify the river continuum. The environmental variables we explored were stream order (stream classification based on size and position in the watershed; Stream Order), bifurcation class (reflects the presence of stream junctions and divergence; BifClass), gradient classification (stream bed slope; GradClass), divergence (describes how a stream segment splits downstream; Divergence), slope of the stream segment (a unitless measure of how steep the stream segment is; Slope), valley confinement (describes how the stream segment relates to the surrounding hill slope; Confinement), elevation (height in meters above sea level of the catchment; ElevCat), percent sand (percentage of sand substrate in the catchment; SandCat), percent clay (percentage of clay substrate in the catchment; ClayCat), rock depth (measure of rock substrate depth in centimeters in the catchment; RckDepCat), and total drainage area (total surface area in square kilometers upstream of a stream‐segment; TotDA) (Table 1; Table S1). We examined correlation among this candidate variable set using Spearman's rank correlation to account for non‐normally distributed data and set a cutoff < 0.7 (Dormann et al. 2013) to avoid potential issues with multicollinearity. Among those that were highly correlated, we removed stream order, bifurcation order, and gradient classification which allowed us to retain variables such as total drainage area, which have been shown to be influential to fish distributions in other research (Bouska et al. 2015; Brewer et al. 2007; Taylor et al. 2017).

**TABLE 1:** Summary of environmental variables for the ALL, ARW, and LMT ranges, reported as the mean (minimum–maximum).

Variable	Description	ALL	ARW	LMT	Removed
Stream Order*	Stream size	2 (1 to 10)	2 (1–9)	2 (1 to 10)	Yes
BifClass*	Bifurcation class	1_0 (0_0 to 9_9)	1_0 (1_0–9_9)	1_0 (0_0 to 9_9)	Yes
GradClass*	Gradient class	Very low (steep–very low)	Moderate (steep–moderate)	Very low (steep–very low)	Yes
Confinement*	Stream divergence	Unconfined (none–unconfined)	Unconfined (moderately confined–unconfined)	Unconfined (none–unconfined)	No
ElevCat (m)	Valley confinement	336.5 (−1.995 to 3981.9)	508.5 (20.1–3981.9)	170.1 (−1.995 to 1747.7)	No
SandCat (%)	Slope	23.0 (1.82 to 82.6)	25.7 (4.60–82.6)	20.5 (1.82 to 80.5)	No
ClayCat (%)	Elevation	30.0 (4.27 to 73.7)	30.1 (4.27–63.9)	29.8 (7.37 to 73.66)	No
RckDepCat (cm)	Percent sand	136.3 (41.3 to 153.8)	129.3 (42.4–152.4)	143.4 (41.3 to 153.8)	No
Divergence	Percent clay	0.585 (0 to 7)	0.426 (0–7)	0.774 (0 to 7)	No
Slope	Rock depth	0.01 (0 to 3.02)	0.01 (0–1.046)	0.01 (0 to 3.02)	No
TotDA (km^2^)	Total drainage area	7691.7 (0 to 3,133,387)	2313.4 (0–39,7422)	12,985.1 (0 to 3,133,387)	No

*Note:* Any variables with high correlation were removed from model analysis. Ordinal variables indicated an asterisk (*) were summarized by mode (range of categories).

### 2.4 Distribution Models

Our modeling approach was to first produce a suite of Maxent models and then select the best performing model for further analysis. This was accomplished through the package ENMeval2.0 v2.0.4, which provides a workflow for tuning model parameters, assessing model performance, and other features for optimizing SDM analyses (Kass et al. 2021). Prior to running our models, we formatted input datasets as samples‐with‐data (SWD; as opposed to raster data) to represent each stream segment by COMID, omitted any stream segments that were represented more than once (whether as an occurrence or background record), and removed stream segments that lacked complete coverage of environmental variables. With ENMeval2.0, users can specify regularization multipliers and feature classes that are involved in model complexity computations (Phillips et al. 2006). We set the models to run with the “maxent.jar” implementation; regularization multipliers (*β*) 1, 2, 3, 4, and 5; and four distinct feature class (*fc*) combinations: linear (L); linear + quadratic (LQ); linear + quadratic + hinge (LQH); and hinge (H) and then explored all combinations of *β* and *fc*. It is important that the models' training data are independent of the testing data to reduce spatial bias and maximize predictive performance (Veloz 2009). We addressed this using a spatial *k*‐fold cross‐validation, which is a reliable approach in cases where there are suspected issues with non‐independent data and when performing model extrapolation (Roberts et al. 2017). We defined spatial partitioning of training and testing bins (folds) based on their location within USGS 8‐digit HUC watersheds (Figure 1C). In these ways, we balanced potential trade‐offs of model complexity and overfitting, while also accounting for the potential effects of spatial bias in model evaluation.

We selected the top ENMeval2.0 models based on a ΔAIC_c_ score of zero (Warren and Seifert 2011) and proceeded with further analysis in R package *dismo* (Hijmans et al. 2017) using the “maxent.jar” implementation. The *dismo* package allowed for more detailed exploration of the top models, as well as projection of models into opposing ranges. The background number was set to 250,000, which allowed all background segments to be used in the ARW and LMT models but required a random subsampling of background for the ALL model. We used jackknife tests to measure variable importance. The raw outputs of suitability were cloglog transformed and joined to NHDPlusV2 stream segments for visualization in ArcMap. To visualize suitability estimates, we used the Minimum Training Presence (MTP; represents the lowest predicted suitability value of an occurrence record) as the minimum suitability cutoff and adopted the following classifications: values of MTP—0 (e.g., 0.218115 for the ALL model) as unsuitable, MTP—0.500000 as low suitability, 0.500001–0.750000 as moderate suitability, and 0.750001–1.0000 as high suitability.

### 2.5 Projections and Ecological Underpinnings

We also used the R package *dismo* to project Maxent models into novel ranges to explore the transferability of the top ARW and LMT models projected into their opposing ranges (i.e., ARW into the LMT range and vice versa). Our projected models were run with ‘clamping’ so that predictions are constrained to environmental conditions of the training data used to calibrate the models. Areas of congruence between in situ and projected models on either side of the Mississippi River would indicate strong agreement regarding suitability of habitat and areas where estimated ecological niche space overlap. Conversely, incongruences could indicate inherent differences in the ecological niches occupied by BFS on either side of the biogeographic divide. Areas projected to be suitable could also assist in identifying habitats where BFS have not been documented but could occur.

Because there is inherent uncertainty associated with projecting models into novel environmental conditions (Barbosa et al. 2016; Elith and Leathwick 2009; Elith et al. 2011), we explored the ecological underpinnings of any incongruence between in situ and projected models on either side of the Mississippi River. Specifically, we asked two different—but inherently interrelated—questions regarding differences in geographic space (G‐space) and environmental space (E‐space; Peterson et al. 2012). First, how different are the environmental conditions on either side of the Mississippi River biogeographic divide, and how might this have affected projected estimates of G‐space? To address this, we used the Multivariate Environmental Similarity Surfaces (MESS) tool from R package *modEVA* to measure similarity between the training data and the predicted range (Barbosa et al. 2016). Our MESS models were developed by including only a subset of the most important environmental variables as determined by Maxent model outputs. MESS analysis produces metrics wherein positive values indicate similarity and negative values indicate dissimilarity (Elith et al. 2010). We mapped MESS results to reference the extent of extrapolation involved with our projected models. Second, how much overlap is there in E‐space given the environmental conditions associated with BFS occurrence records on either side of the divide? To address this question, we used the R package *hypervolume* to visualize ARW and LMT niche hypervolume in E‐space and calculate metrics of overlap (Jaccard and Sørensen similarities) and fractions of each unique hypervolume (Blonder et al. 2014). We again used only the top‐contributing variables from Maxent models but transformed them with *z*‐scores (prior to splitting them into ARW and LMT ranges) to account for potential biases in hypervolume estimation that can be introduced when incorporating dissimilar variables (Blonder et al. 2014). Examining these two questions provided additional ecological context to modeling results.

## 3 Results

### 3.1 Model Inputs

There was a total of 762 BFS occurrences linked to 508 occurrence stream segments (repeat records at the same location were treated as one stream segment in modeling). When split by the eastern and western ranges, there were 431 BFS occurrences linked to 225 occurrence stream segments within the ARW and 331 BFS occurrences linked to 283 occurrence stream segments in the LMT. There was a total of 391,361 background stream segments (ALL), with 185,507 background stream segments in the ARW and 197,240 stream segments in the LMT. The earliest BFS record was from the year 1911 and the most recent was from 2018. Environmental variable ranges differed noticeably between the ARW and LMT (Table 1).

### 3.2 Model Fitting and Evaluation Metrics

The best‐fit model for the ALL range involved an *fc* combination of LQH, and a *β* of 4. The best‐fit model for the ARW range involved an *fc* combination of LQH and *β* of 3. The best‐fit model for the LMT involved an *fc* combination of LQH and *β* of 2 (Table 2). Overall, evaluation metrics indicated models had strong predictive power without overfitting as area under the curve (AUC) values approached 1 and omission rates of training data were near the 10% evaluation target for each model.

**TABLE 2:** Model metrics for the first five ARW and LMT models.

fc	*β*	auc.train	auc.diff.avg	or.10p.avg	or.10p.sd	or.mtp.avg	or.mtp.sd	AIC_c_	ΔAIC_c_	w.AIC	ncoef
ALL											
LQH	4	0.90103	0.0689	0.14686	0.22269	0.0024706	0.01251	11,960	0.0000	1.00	83
LQH	1	0.91364	0.06351	0.17416	0.25387	0.0024706	0.01251	11,975	15.803	0.00	144
LQH	2	0.9097	0.06443	0.17493	0.25353	0.0016026	0.011103	11,982	22.144	0.00	127
H	1	0.91362	0.06338	0.175	0.25389	0.0024706	0.012514	12,000	40.071	0.00	150
LQH	3	0.90565	0.06692	0.17306	0.25427	0.0024706	0.012514	12,001	41.256	0.00	115
ARW											
LQH	3	0.94154	0.08145	0.17676	0.29905	0.00644	0.02270	4621.2	0.0000	1.0000	54
H	3	0.94370	0.07902	0.19949	0.30394	0.05189	0.21297	4641.8	20.537	0.0000	60
LQH	4	0.93844	0.08215	0.20165	0.30439	0.00644	0.02270	4658.1	36.878	0.0000	54
LQH	5	0.93653	0.08096	0.20018	0.30421	0.00644	0.02270	4666.1	44.910	0.0000	47
H	4	0.94172	0.07850	0.20165	0.30439	0.05189	0.21297	4708.8	87.590	0.0000	69
LMT											
LQH	2	0.93847	0.03538	0.13957	0.22086	0.00641	0.03269	6064.7	0.0000	1.0000	64
H	3	0.93670	0.03579	0.13573	0.21327	0.00641	0.03269	6090.3	25.607	0.0000	65
H	2	0.93818	0.03544	0.14198	0.22175	0.00641	0.03269	6091.7	27.066	0.0000	72
LQH	5	0.93315	0.03786	0.12788	0.20833	0.00641	0.03269	6099.7	35.034	0.0000	52
LQH	3	0.93703	0.03620	0.13188	0.20725	0.00641	0.03269	6109.2	44.483	0.0000	70

*Note:* Model parameters are represented by feature class (fc) combinations, regularization multiplier (*β*), and coefficients (ncoef). Top models were selected based on Akaike information criterion (AIC), but other model performance metrics used as references included: Area under curve training values (auc.train), the average difference (auc.diff.avg), and 10% omission rate (or.10p).

### 3.3 Estimated Distributions

**FIGURE 2:**
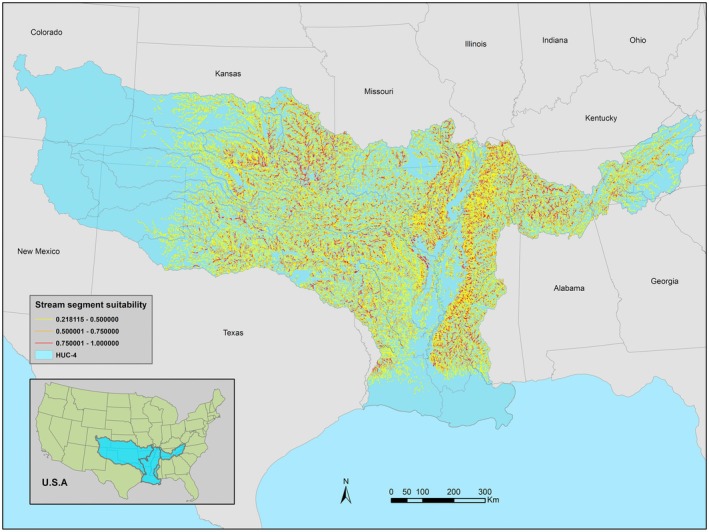
ALL model estimated stream segment suitability with occurrence records of Bluntface Shiner and environmental variables from the Arkansas‐Red‐White, Lower Mississippi, and Tennessee river basins.

The ALL model estimated an expansive distribution that spanned from the central watersheds of the Arkansas‐Red‐White to the parts of the coastal watersheds of the Lower Mississippi and the far eastern watersheds of the Tennessee River Basin (Figure 2). Most watersheds had stretches of moderate to highly suitable stream segments separated by areas of low to unsuitable stream segments. Some notable areas of highly suitable habitats included portions of the Neosho, Verdigris, and Arkansas watersheds within the ARW, and the Pearl, Big Black, White, and lower Tennessee watersheds within the LMT.

**FIGURE 3:**
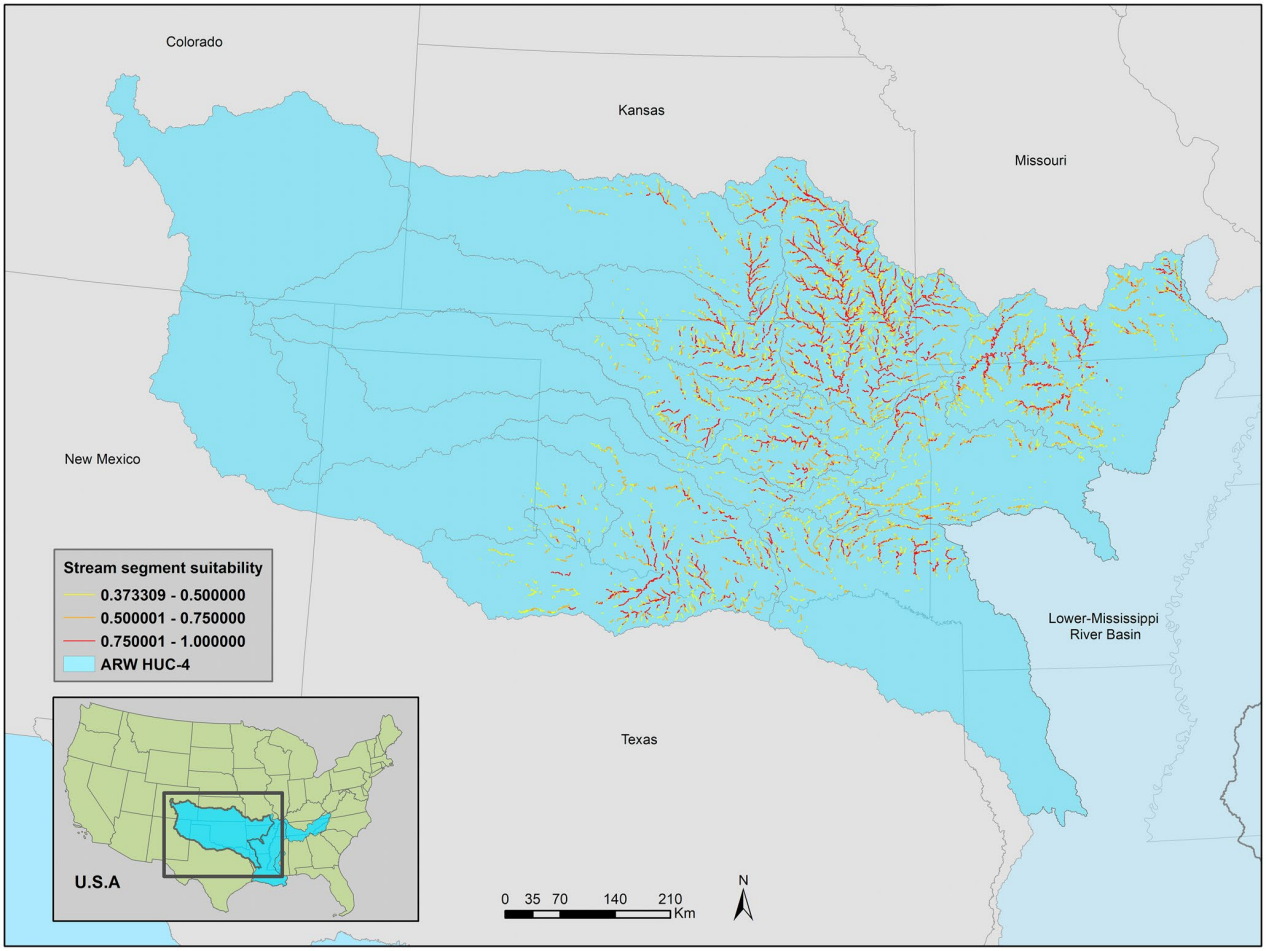
ARW model estimated stream segment suitability with occurrence records of Bluntface Shiner and environmental variables of the Arkansas‐Red‐White river basin.

The ARW model estimated a distribution confined to the central portion of the previously documented range (Figure 3). Like the ALL model, the Neosho and Verdigris watersheds had moderate to highly suitable stream segments across the entire watershed. Watersheds along the Red, Canadian, Cimarron, Arkansas, and Upper‐White had some highly suitable stream segments but were mostly of low suitability or unsuitable. The westernmost watersheds and those nearest the lower Mississippi River basin were estimated to be unsuitable for BFS (Figure S1).

**FIGURE 4:**
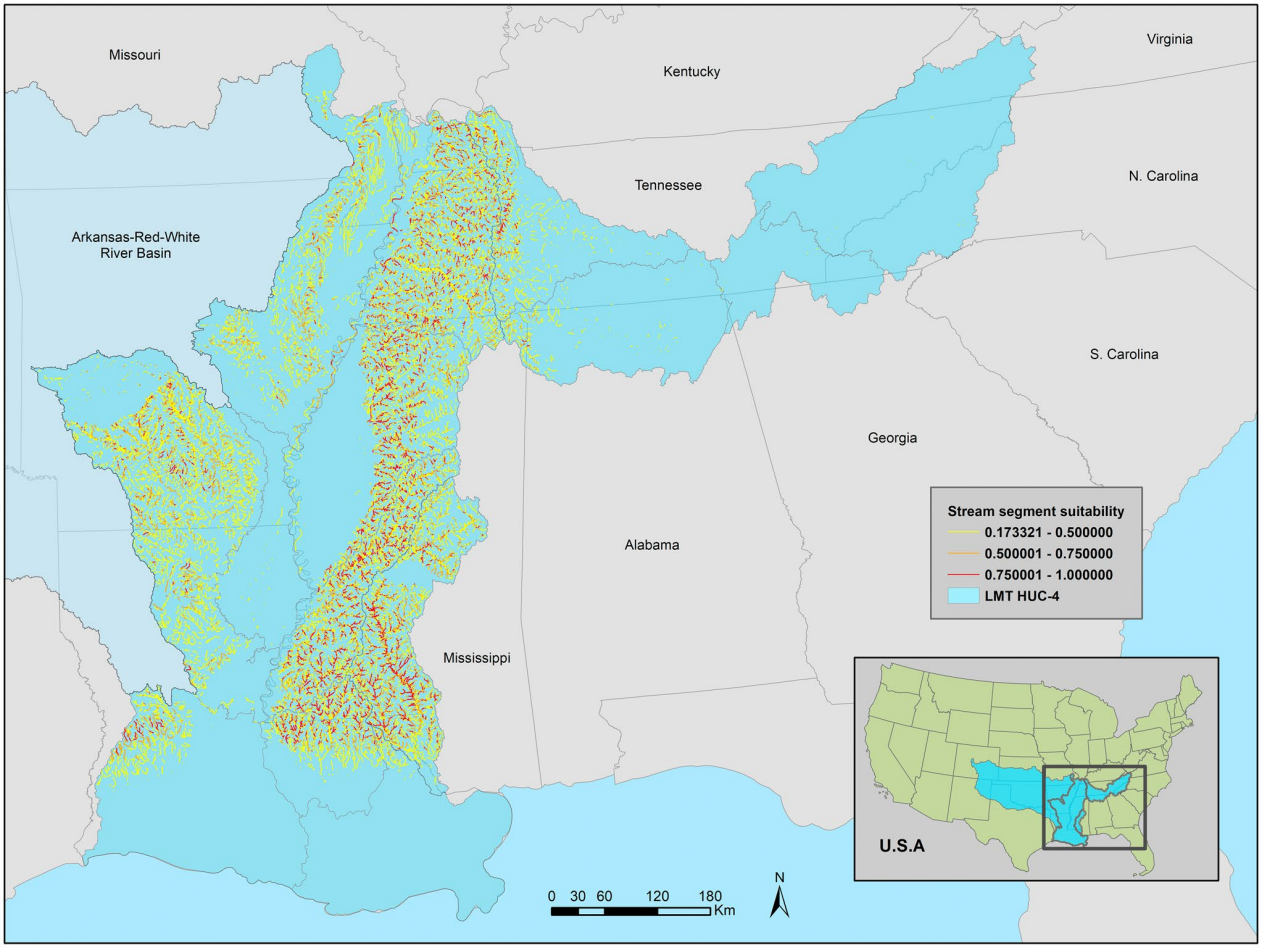
LMT model estimated stream segment suitability with occurrence records of BFS and environmental variables of the Lower Mississippi and Tennessee river basins.

The LMT model estimated a narrow distribution within the previously documented range (Figure 4). Watersheds with the highest concentration of highly suitable segments were those east of the Mississippi River, such as the Hatchie, Big Black, and Pearl. The Yazoo and Lake Maurepas watersheds also held highly suitable segments but only in discrete portions of those watersheds. Coastal watersheds, portions of watersheds along the western side of the lower Mississippi River, and the majority of the Tennessee River basin were estimated as unsuitable.

### 3.4 Response Curves

There were eight environmental variables (ElevCat, SandCat, ClayCat, RckDepCat, Slope, Divergence, Confinement, and TotDA) that informed our models. The top model for each range contained a slightly different combination of variables with the highest percent contribution and permutation importance (Table 3). In the ALL, ARW, and LMT models, the variable TotDA contributed the most to model predictive performance, followed by the remaining variables in a different order for each model. Hereafter, we compare variables of the top models that had a percent contribution or permutation importance value above 10. Importantly, percent contribution provides a relative measure of increase in model predictive ability, but this does not equate to ecological importance because of confounding factors such as varying scales of magnitude or natural outliers in environmental variables.

**TABLE 3:** Highest ranked environmental variables determined by percent contribution (Contr. %) and permutation importance (Perm. imp.).

Variable	ALL		ARW		LMT	
	Contr. %	Perm. imp.	Contr. %	Perm. imp.	Contr. %	Perm. imp.
TotDA*	54.4	48.3	47.1	46.2	36	37.8
ElevCat*	20.1	17.3	19.3	34.6	6.7	10.6
ClayCat*	12	21.1	15.7	8.1	28.8	34.2
RckDepCat*	0.1	0.1	6.1	2.6	13.9	6.4
SandCat	3.8	3.7	6.3	5.5	1.4	1.8
Slope	3.8	4.7	4.9	2.2	3.9	2.6
Divergence	5.8	4.7	0.5	0.2	7.4	5.5
Confinement	0	0	0.2	0.6	1.8	1.1

*Note:* Variables with values > 10 in at least one model are highlighted with an asterisk.

**FIGURE 5:**
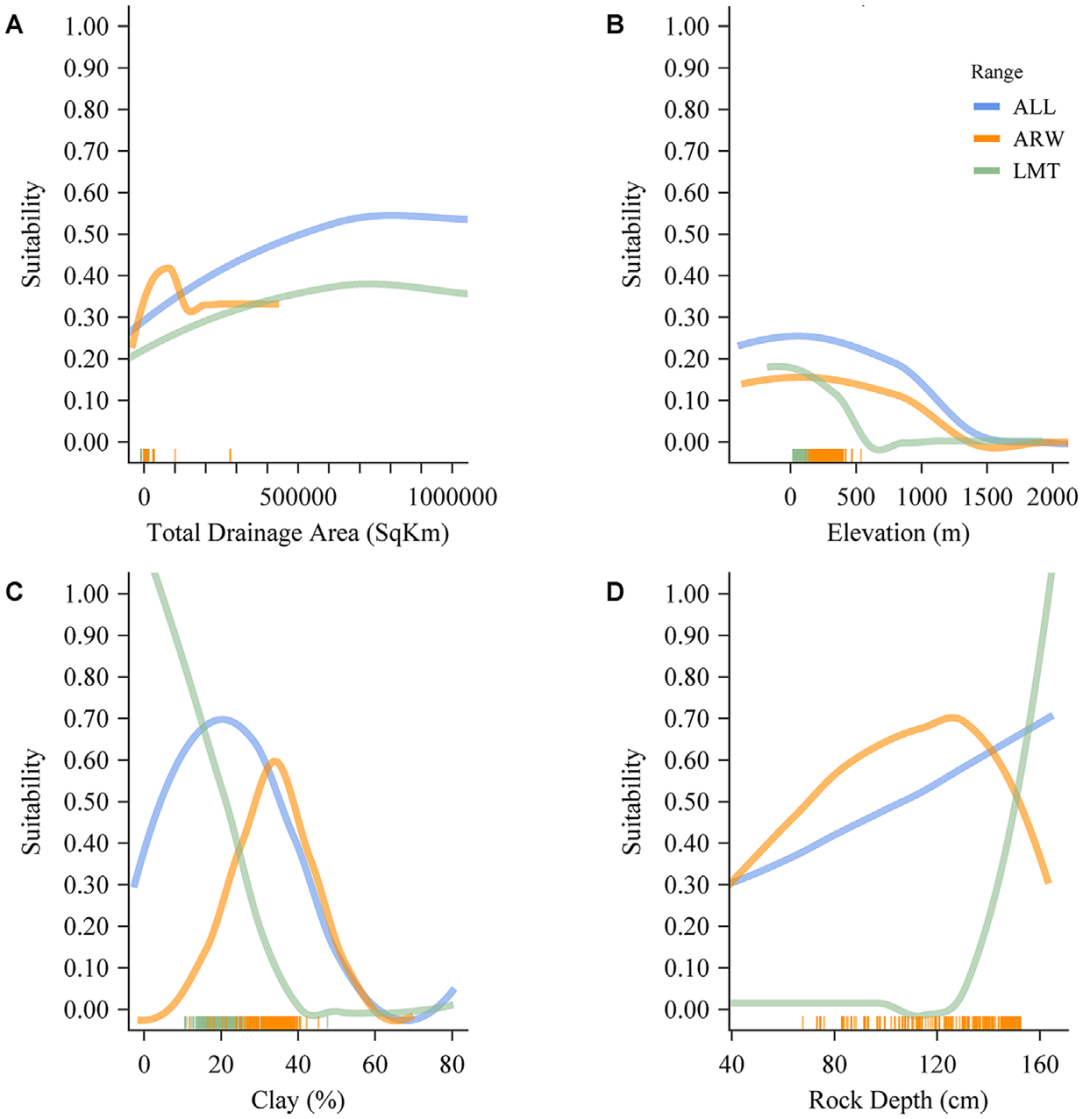
Response curves depicting the highest contributing environmental variables plotted against predicted suitability values of BFS for the ALL, ARW, and LMT ranges. Occurrence records corresponding with the curves are represented along the *x*‐axis (tick marks).

In each of our models, total drainage area had the highest percent contribution. In the ALL and LMT ranges, there was a gradual increase in suitability with increased total drainage sizes. In the ARW range, the response curve was unimodal with the highest suitability at ~100,000 km^2^ (Figure 5A). Elevation was the second highest contributing variable for the ALL and ARW ranges, though it was not important in the LMT. In the ALL and ARW ranges, the response curves had slight increases at low elevations before gradually decreasing at higher elevations. In the LMT range, the response curve had its highest suitability at low elevations before sharply decreasing in suitability at higher elevations (Figure 5B). Clay percentage within the catchment was the third highest contributing variable for the ALL and ARW ranges and was the second highest contributing variable for the LMT range. In the ALL range, the response curve was unimodal with the highest suitability at ~20%. The ARW range response curve was also unimodal with suitability that peaked at ~40%. In the LMT range, suitability had a sharp decline as percent clay reached ~40% (Figure 5C). Rock depth was the third highest contributing variable for the LMT range while contributing little to the ALL and ARW ranges. In the LMT range, the response curve had a sharp increase at rock depths > 120 cm, whereas the ALL range had a gradual increase in suitability as rock depth increased. Meanwhile, the ARW range had a unimodal response curve with suitability increasing to its maximum at ~130 cm before declining at greater depths (Figure 5D).

### 3.5 Projections and Ecological Underpinnings

**FIGURE 6:**
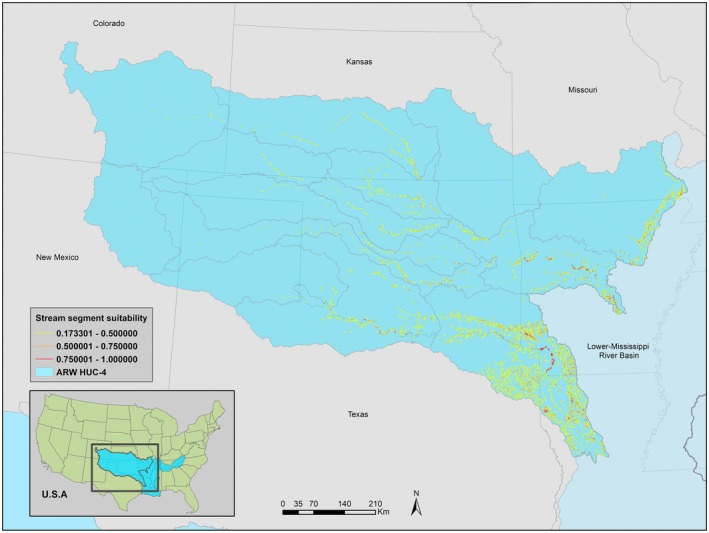
LMT model estimated stream segment suitability projected into the Arkansas‐Red‐White basin.

**FIGURE 7:**
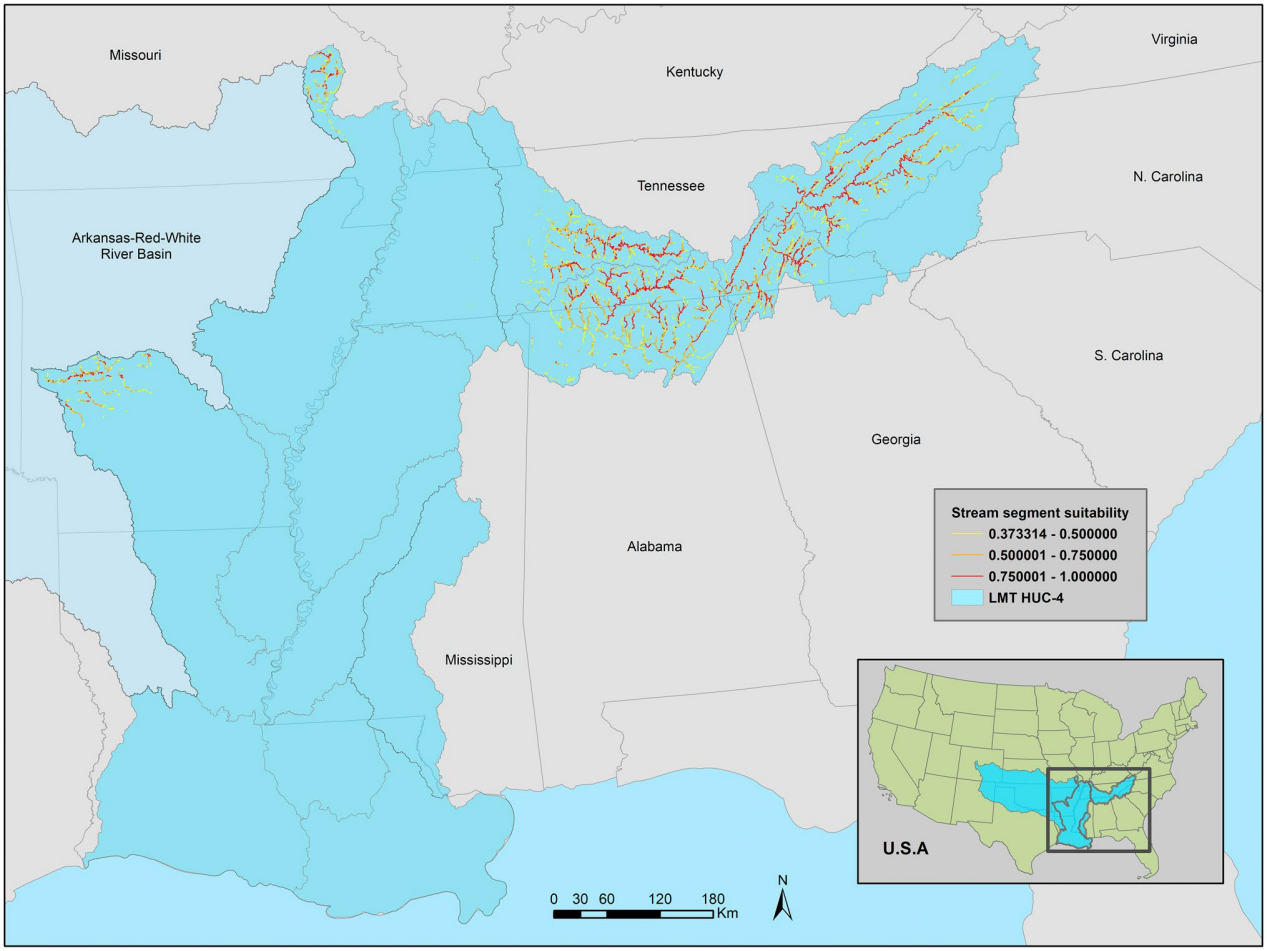
ARW model estimated stream segment suitability projected into the Lower Mississippi and Tennessee river basin.

We projected the ARW and LMT models into opposing ranges, and in both cases, there were considerable range shifts compared to the known occurrences and estimated ranges from in situ Maxent models. When the LMT model was projected into the ARW range, the estimated range shifted to the extreme southeastern edge of the ARW into regions that BFS are not known to occur (Figure 6). There were some low suitability stream segments that stretched into the center of the ARW region; however, most of the higher suitability stream segments were along the border with the lower Mississippi River Basin. When the ARW model was projected into the LMT range, suitable segments were confined to small areas to the extreme northeast of the lower Mississippi River basin (Figure 7). Unlike the in situ LMT model and existing BFS occurrence records, the ARW projection resulted in expansive stretches of highly suitable stream segments within the Tennessee River basin. Save for the coastal watersheds and along the lower Mississippi River, estimates of suitable habitat were nearly inverted when the ARW was projected into the LMT (Figure S2).

The MESS analysis illuminated similarities in G‐space that may help explain the drastic shift in the estimated suitable range for BFS when projecting models into opposing ranges. When extrapolating the LMT model into the ARW range, stream segments with highest similarity (most positive MESS values) were scarce and only existed along the southeastern edge of the ARW nearest to the lower Mississippi River basin (Figure S3). When extrapolating the ARW model into the LMT, stream segments with highest similarity were also scarce and mostly found within the Tennessee River Basin (Figure S4). From an E‐space perspective, visualization of a four‐dimensional niche hypervolume illustrated some appreciable differences. Most notably, BFS in the LMT occupied areas with a combination of lower elevation and lower clay in the catchment than did BFS in the ARW (Figure S5). Metrics of niche similarity were extremely low (Jaccard = 0.0001 [95% CI 0.0000–0.0005]; Sørensen = 0.0003 [95% CI 0.0000–0.0009]), whereas the fractions of unique hypervolume space for each range were high, with ARW at 0.9999 (95% CI 0.9995–1.0000) and LMT at 0.83333 (95% CI 0.68889–1.0000).

## 4 Discussion

Species distribution models (SDMs) allow investigators to estimate a geographic distribution and portions of the ecological niche that underlie the distribution, both of which can inform ecology and conservation (Cooke et al. 2012; Graham et al. 2004; Matthews 1998). SDMs like Maxent are advantageous because they use readily available presence‐only data and allow users to tune models to address sample selection bias and optimize model performance (Dudik et al. 2004; Phillips and Dudík 2008). We used SDMs to investigate the Bluntface Shiner (BFS), a minnow that is likely declining across large portions of its range, and whose previously described ecology largely centered on microhabitat use. Our modeling revealed broadscale species–habitat relationships and estimated where BFS may occur while accounting for potential ecological differences of BFS to the east and west of the Mississippi River, a prominent biogeographical divide for freshwater fishes. Furthermore, projections of models constructed from their disjunct ranges demonstrated large differences in the environmental conditions that BFS occupy on either side of this biogeographic divide. Although our findings will inform future ecological study and conservation action, our results also raise additional questions regarding the taxonomy of BFS and the potential ramifications that could have on conservation status.

Our models captured various stream network features that are influential to fish occurrence and potentially important for BFS population dynamics. For example, the highest contributing variable across the ALL, LMT, and ARW models was total drainage area. This variable may be important for various reasons because drainage area is associated with factors including water volume and discharge, stream depth, size, length, and various physicochemical properties that change with increasing size (Allan et al. 2021; Matthews 1998; Matthews and Robison 1998). Our results concur with previous reports that BFS are most associated with medium to large‐sized streams (Cross 1954; Etnier and Starnes 1993; Farr 1996; Wilkinson and Edds 2001). Larger drainage areas likely provide BFS with greater access to important spawning, rearing, and refuge habitat, as well as opportunities for dispersal and recolonization.

Our suitability maps provided a unique characterization of the species distribution, and we were able to identify several consistently suitable and unsuitable watersheds for BFS in both their ranges. For example, the Neosho and Verdigris rivers to the west and the Pearl River to the east consistently held highly suitable stream segments, while the lower Mississippi River was consistently unsuitable. In fact, our models suggest the habitats of the lower Mississippi River are largely unsuitable, resulting in disjunct patches of suitable habitats for BFS to the east or west. This is potentially valuable information for biogeographic and phylogenetic investigations as investigators can better understand contemporary distributions by reconstructing the dispersal and diversification of riverine fishes through geologic time (Strange and Brooks 1997).

Within the LMT and ARW ranges, a glaring feature of estimated distributions was that suitable streams for BFS were disjoined and relatively limited in quantity. This patchy matrix of suitable habitats was not entirely surprising given that riverine ecosystems naturally vary in physical, hydrological, and chemical conditions as they flow from headwaters to the river mouth (Hynes 1975; Vannote et al. 1980), and stream segments may have distinct physical conditions from adjacent segments (Poole 2010). However, the naturally fragmented distribution of suitable habitats suggests that BFS are susceptible to local population declines or extirpations (e.g., Fagan et al. 2005, 2002). Such concerns are heightened when also considering anthropogenic alteration of these riverscapes. The ARW and LMT have experienced major alteration over the past 100 years, particularly from the construction of dams, levees, and reservoirs along with increasing urban and agricultural land uses (Pennock 2017; Remo et al. 2018, 2009; Simon et al. 2020; Turner and Rabalais 2003; Yang et al. 2023). These factors alter fish species distributions through physical blockage of movement (i.e., migration or recolonization) and by deleterious changes to habitat, which together harm sensitive species like BFS (Jester et al. 1992). Consequently, the contemporary distribution of BFS in the ARW and LMT is likely more restricted and fragmented than our potential distribution estimates.

The disjunct BFS range presented an interesting opportunity to test model performance when models were projected into opposing ranges across the Mississippi River. We expected some overlap in suitable conditions between the LMT and ARW given similarities in previously reported microhabitat requirements of BFS and similarities in the percent contributions of our environmental variables across models. In fact, we hoped that projecting distributions across the Mississippi River might identify novel stream systems to survey for BFS. However, to our surprise, the projected models resulted in severe mismatches with known occurrences and in situ estimates of distributions. Indeed, many SDM studies emphasize the need for caution when extrapolating models across vast distances for both methodological and ecological reasons (Elith and Leathwick 2009; Werkowska et al. 2017). In our study, ecological differences quantified by complementary analyses of G‐space and E‐space suggest that most of the mismatch between in situ and projected distributions can be attributed to considerable differences in the occupied environmental conditions between the ARW and LMT. Although BFS from either range are said to have similar microhabitat associations (Distler et al. 2014; Etnier and Starnes 1993; Ross and Brenneman 2001), our results suggest that BFS east and west of the lower Mississippi River occupy largely different habitat at riverscape scale.

The marked differences in occupied environmental conditions of BFS on either side of the biogeographic divide of the Mississippi River could have a couple of different eco‐evolutionary interpretations. First, from a Hutchinsonian viewpoint, it could be posited that all occupied conditions on either side of the divide capture elements of the fundamental niche of BFS, whereas factors such as interspecific competition that vary geographically may result in different realized niches. For example, competition and hybridization with Red Shiner ( *C. lutrensis* ) (Cross and Cavin 1971; Johnston 1999) could largely exclude BFS from lower elevation streams within the ARW that would otherwise be environmentally similar to conditions BFS occupy in the LMT. Conversely, our results add ecological support to a growing body of evidence that suggests BFS on either side of the Mississippi River may, in fact, represent two distinct species. Early investigations of morphological distinctiveness (Gibbs Jr. 1961) and genetic studies (Mayden 1989) both discovered some degree of differentiation between populations of the ARW and LMT. Moreover, phylogenetic analysis using *cyt b* sequences of BFS in the ARW and LMT revealed the disjunct populations as two distinct lineages (Egge and Hagbo 2015), adding further credence to the idea of a unique species designation. Given the previous phenotypic and molecular evidence, coupled with our findings of relatively distinct ecological niches, a precautionary approach may be warranted in which the ARW and LMT populations are viewed as distinct units for the purposes of biodiversity conservation, at least until a more thorough and conclusive taxonomic investigation is completed.

Knowledge of species distributions and underlying habitat relationships is fundamental to better ecological understanding and conservation of species (Elith et al. 2006), yet many threatened riverine fishes suffer from a lack of information regarding these key aspects (Cooke et al. 2012). Species distribution models are particularly useful for addressing such knowledge gaps, and our models reinforced this utility. Current knowledge of BFS life history is restricted to microhabitat associations, and while this information is valuable, the effectiveness of conservation and management actions is maximized when processes acting at broader spatial scales are incorporated (Roni et al. 2008). Along these lines, our analysis included both disjunct distributions of BFS, and we revealed important landscape‐scale features that characterized the potential distributions in both ranges; therefore, these findings have considerable applications for conservation and management of BFS. For example, the distribution maps can be used as a baseline to gauge the extent of range loss in areas where BFS have experienced declines and identify at‐risk populations most in need of monitoring and management. Our distribution maps revealed hotspots of suitability that could help narrow down search efforts for extant BFS populations, as well as identify areas of high conservation priority where preserving habitats may be most critical. Likewise, determining locations with robust BFS populations provides opportunities to investigate life‐history aspects such as reproductive ecology. Similarly, impaired watersheds that hold suitable habitats could present opportunities for habitat restoration and potential repatriation efforts.

Despite several useful applications of the SDMs and projections we conducted, we acknowledge that our models likely represent an optimistic approximation of contemporary BFS distribution, and some streams estimated to be suitable may not be in reality. The presence‐only nature of Maxent models does not consider true absence data, nor information on the abiotic or biotic factors that preclude the occurrence of BFS at finer spatial scales. Indeed, presence–absence data typically provide more robust model predictions (Yackulic et al. 2013). However, the problem of imperfect detection, or potentially not detecting a target species although it is there (i.e., a “false absence”), should be accounted for to avoid biases in model outputs and interpretation. Imperfect detection is a common issue when sampling for rare or difficult‐to‐detect stream fishes such as BFS, but can be accounted for with intensive standardized surveys and occupancy modeling frameworks designed to account for variables that affect underlying species detection rates (Dextrase et al. 2014; McManamay et al. 2014). Future research could focus on estimating detection and occupancy probabilities for BFS to better understand factors that determine their presence and absence, as well as how to most efficiently survey them. Such investigations could also consider anthropogenic land use and river modifications (e.g., agriculture and urbanization, dams and impoundments), as these are pervasive features across the contemporary distribution of BFS.

## Author Contributions

**Briant D. Nguyen:** conceptualization (equal), data curation (lead), formal analysis (lead), investigation (equal), methodology (lead), writing – original draft (lead), writing – review and editing (lead). **Jenna Messick:** conceptualization (equal), formal analysis (equal), investigation (equal), methodology (equal), writing – original draft (equal), writing – review and editing (equal). **Anthony W. Rodger:** conceptualization (equal), formal analysis (equal), investigation (equal), methodology (equal), writing – original draft (equal), writing – review and editing (equal). **Victoria Jackson:** conceptualization (equal), formal analysis (equal), investigation (equal), methodology (equal), project administration (supporting), supervision (equal), writing – original draft (equal), writing – review and editing (equal). **Christopher Butler:** conceptualization (equal), formal analysis (equal), funding acquisition (lead), investigation (equal), methodology (equal), project administration (equal), writing – original draft (equal), writing – review and editing (equal). **Andrew T. Taylor:** conceptualization (lead), data curation (equal), formal analysis (equal), funding acquisition (lead), investigation (equal), methodology (equal), project administration (lead), supervision (lead), writing – original draft (equal), writing – review and editing (equal).

## Conflicts of Interest

The authors declare no conflicts of interest.

## Supporting information


Appendix S1

## Data Availability

A supplemental file accessible with the online version of this manuscript contains the Supporting Information Tables and Supporting Information Figures referenced herein. Input data files and R code to reproduce data analyses and visualizations are available on GitHub: https://github.com/ATaylorFish/BluntfaceShiner_Distribution (and are included for peer review as a downloadable attachment).
