# Supplementary material for: Lumping and Splitting of Distribution Models Across a Biogeographic Divide Informs the Conservation of an Imperiled Fluvial Fish
Source: Ecol Evol. 2025 Apr 25;15(4):e71315. doi: 10.1002/ece3.71315 (PMC12032192; doi:10.1002/ece3.71315)
Supplement: Supplementary file 1 — Appendix S1 [file ECE3-15-e71315-s001.docx]

Supplemental materials for

**Lumping and splitting of distribution models across a biogeographic divide informs the conservation of an imperiled fluvial fish**

Briant D. Nguyen^1^, Jenna Messick^1^, Anthony W. Rodger^2^, Victoria Jackson^1^, Christopher Butler^1,3^, Andrew T. Taylor^1,4^

1 Department of Biology, University of Central Oklahoma, Edmond, OK, USA

2 Oklahoma Department of Wildlife Conservation, Stream Program, Porter, OK, USA

3 Current Address: Department of Biology, Texas A&M University, College Station, TX, USA

4 Current Address: Department of Biology, University of North Georgia, Dahlonega, GA, USA; ORCID: <https://orcid.org/0000-0002-8491-9967>

**This document includes:**

ODMAP Protocol

Tables S1

Figures S1 - S5

**ODMAP Protocol –**

**Overview**

*Model objective*

Model objective: Mapping and interpolation, projection and extrapolation

Target output: Continuous habitat suitability index

*Focal Taxon*

Focal Taxon: Bluntface Shiner (Cyprinella camura)

*Location*

Location: Arkansas, Red, White, Lower Mississippi, and Tennessee river basins

*Scale of Analysis*

Spatial extent: Arkansas-Red-White (HUC-11), Lower-Mississippi (HUC-08), and Tennessee (HUC-06)

Spatial resolution: Stream segment

Temporal extent: n/a

Boundary: natural

*Biodiversity data*

Observation type: field survey

Response data type: presence-only

*Predictors*

Predictor types: habitat, topographic

*Hypotheses*

Hypotheses: The disjunct Bluntface Shiner populations share similar environmental conditions on either side of the Lower Mississippi River

*Assumptions*

Model assumptions: Study species is at equilibrium with the environment. Occurrence data collection is spatially independent

*Algorithms*

Modelling techniques: maxent

Model complexity: Maxent allows for model tuning to balance model complexity and over fitting

Model averaging: AICc (Akaike Information Criterion corrected) was used to determine the best performing model

*Workflow*

Model workflow: Our first step was to create a suite of distribution models for both disjunct ranges individually and combined as one. Then, select the highest performing model for each range combination based on delta AICc score of zero. We used k-fold cross-validation with spatial partitioning to address potential issues with spatial bias in the data. Model performance was assess using AUC and omission rate metrics. With the best model, we project models of the western estimated distribution into the eastern range and the eastern estimated distribution into the western range. Clamping was done with model projection. To further explore differences between models, we used Multivariate Environmental Similarity Surfaces and hypervolume analysis. Jackknife test was used to assess most important environmental predictor. Model outputs were cloglog transformed for suitability values that were used for mapping.

*Software*

Software: R coding language v4.1.2 with packages: ENMeval2.0 dismo rmaxent modEVA hypervolume

Code availability: <https://github.com/ATaylorFish/BluntfaceShiner_Distribution>

Data availability: <https://github.com/ATaylorFish/BluntfaceShiner_Distribution>

**Data**

*Biodiversity data*

Taxon names: Bluntface Shiner (*Cyprinella camura*)

Taxonomic reference system: n/a

Ecological level: species, populations

Data sources: GBIF (<https://www.gbif.org>) Fishnet2 (<http://www.fishnet2.net>) IDigBio (<https://www.idigbio.org>) iNaturalist (<https://www.inaturalist.org>) BISON (<https://bison.usgs.gov> Oklahoma Department of Wildlife Conservation Oklahoma Conservation Commission and University of Oklahoma Sam Noble Museum Oklahoma State University

Sampling design: n/a

Sample size: n/a

Absence data: Absence data for this species has yet to be established

Background data: n/a

*Data partitioning*

Training data: spatial partitioning of data based on location with HUC-8 watersheds

Validation data: k-fold cross validation with spatial partitions

*Predictor variables*

Predictor variables: Stream order, bifurcation order, gradient classification, divergence, slope, valley confinement, elevation, percent sand, percent clay, rock depth, and total drainage area

Data sources: NHDplusV2 (<https://www.epa.gov/waterdata/get-nhdplus-national-hydrography-dataset-plus-data>) StreamCat (<https://www.epa.gov/national-aquatic-resource-surveys/streamcat-dataset>) Stream Classification System (<https://www.nature.com/articles/sdata201917>)

Spatial extent: Arkansas-Red-White (HUC-11), Lower-Mississippi (HUC-08), and Tennessee (HUC-06)

Spatial resolution: Watershed, catchment, stream-segment scales

Coordinate reference system: n/a

Temporal extent: n/a

*Transfer data*

Data sources: Transfer data involved the same data used in the potential distribution models

Spatial extent: n/a

Spatial resolution: n/a

Temporal extent: n/a

Models and scenarios: The estimated distribution of the western BFS population was transferred onto the eastern range and vice versa

Quantification of Novelty: Rug plot, MESS, and hypervolume analyses was done to quantify differences in environmental conditions

**Model**

*Multicollinearity*

Multicollinearity: We tested for multicolinearity using Spearman’s rank correlation to account for non-normally distributed data and set a cut off for correlation coefficient r < 0.7 to avoid potential issues with multicollinearity between the variables.

*Model settings*

Maxent:

"betamultiplier=4”

“removeDuplicates=TRUE”

"linear=TRUE"

"quadratic=TRUE"

“product=FALSE"

“threshold=FALSE"

“hinge=TRUE"

“jackknife=TRUE"

Model settings (extrapolation): Clamping was used for projected models

*Model estimates*

Coefficients: n/a

*Analysis and Correction of non-independence*

Spatial autocorrelation: k-fold cross validation

*Threshold selection*

Threshold selection: n/a

**Assessment**

*Performance statistics*

Performance on training data: AUC

Performance on validation data: omission rate (OR), CBI, AUC

Performance on test data: omission rate (OR), CBI, AUC

*Plausibility check*

Response shapes: n/a

Expert judgement: map display

**Prediction**

*Prediction output*

Prediction unit: cloglog for suitability index between 0-1 with 0 being least suitable and 1 being most suitable

*Uncertainty quantification*

Scenario uncertainty: n/a

Novel environments: n/a

| **Table S1.** Environmental variables related to Bluntface Shiner biology were selected from various datasets (NHDPlusv2, StreamCat, Stream Classification System) and linked to all stream segments in the study area. | | | | | |  |
| --- | --- | --- | --- | --- | --- | --- |
| Environmental variables | | | | | |  |
| Abreviation | Description | Unit | Scale | Source | Data Type |  |
| Stream order | Stream size | - | Segment | NHDPlusV2 | Factor |  |
| BifClass | Bifurcation class | - | Segment | SCS | Factor |  |
| GradClass | Gradient class | - | Segment | SCS | Factor |  |
| Divergence | Stream divergence | - | Segment | SCS | Factor |  |
| Confinement | Valley confinement | - | Segment | SCS | Factor |  |
| Slope | Slope | - | Segment | SCS | Continuous |  |
| ElevCat | Elevation | m | Catchment | StreamCat | Continuous |  |
| SandCat | Percent sand | % | Catchment | StreamCat | Continuous |  |
| ClayCat | Percent clay | % | Catchment | StreamCat | Continuous |  |
| RckDepCat | Rock depth | cm | Catchment | StreamCat | Continuous |  |
| TotDA | Total drainage area | km^2^ | Watershed | NHDPlusV2 | Continuous |  |


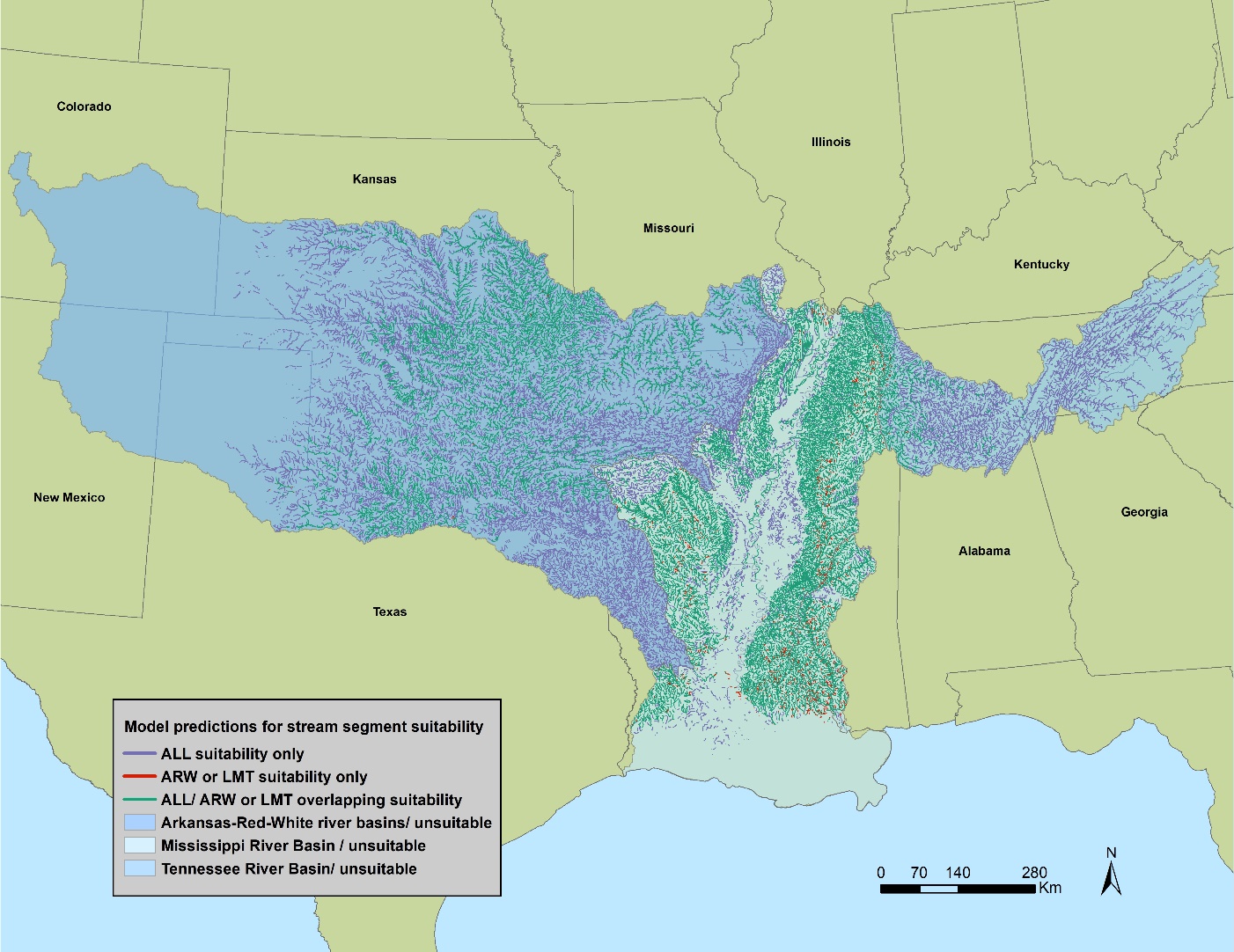


**Figure S1.** Model estimated stream segment suitability using occurrence records of BFS and environmental variables across the study range. The estimated suitable stream segments of the ALL model (dark blue) were overlain with the ARW and LMT models to demonstrate overlap (dark green) and nonoverlap (red) between model’s estimated suitability.


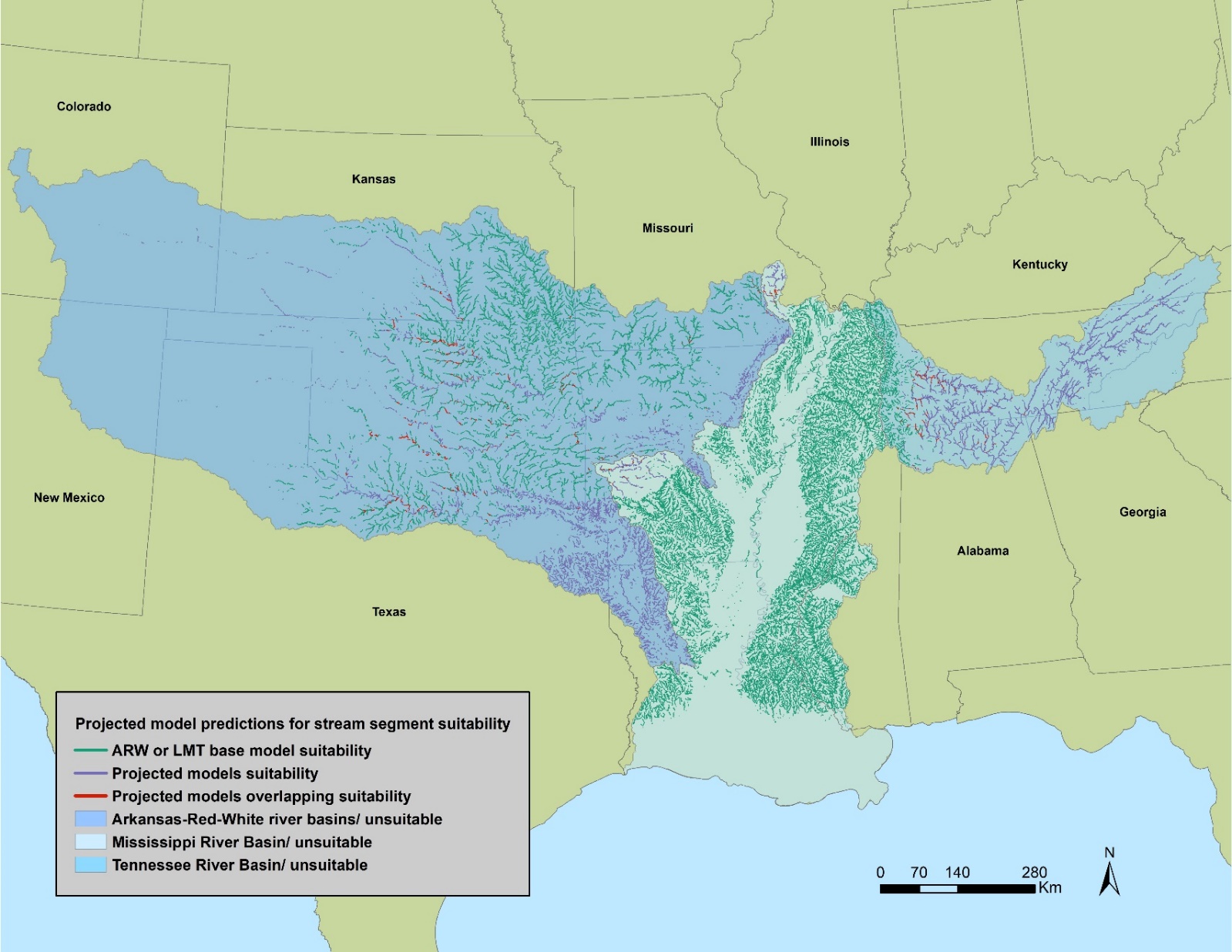


**Figure S2.** The ARW and LMT model’s estimated stream segment suitability when projected onto opposing ranges (dark blue). The base model estimates (dark green) were overlain with the projected models to demonstrate overlap in estimated stream segment suitability (red).


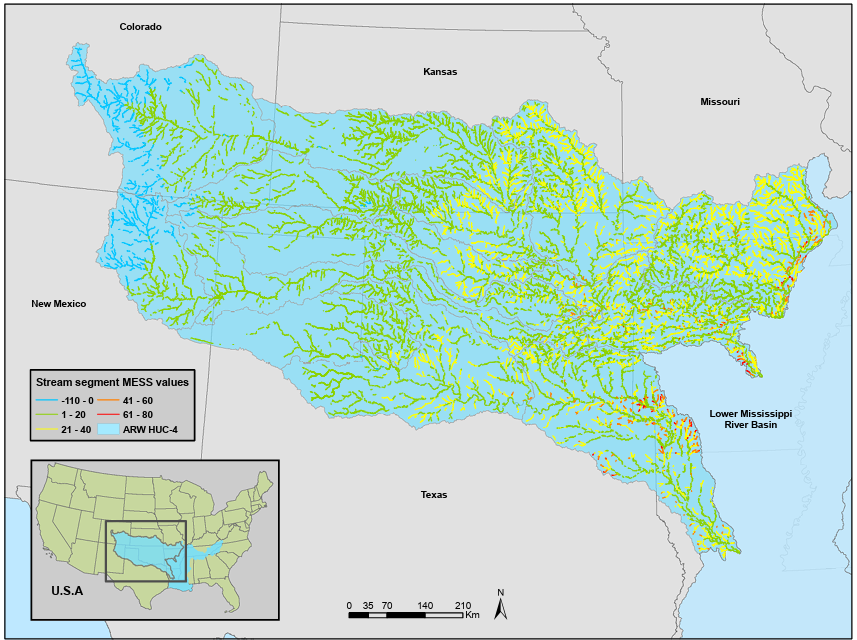
**Figure S3.** Multivariate environmental similarity surfaces (MESS) of the LMT model extrapolated into the ARW range showing similarity (positive MESS values) and dissimilarity (negative MESS values) of LMT stream segments in relation to ARW stream segments (stream order ≥3).

=
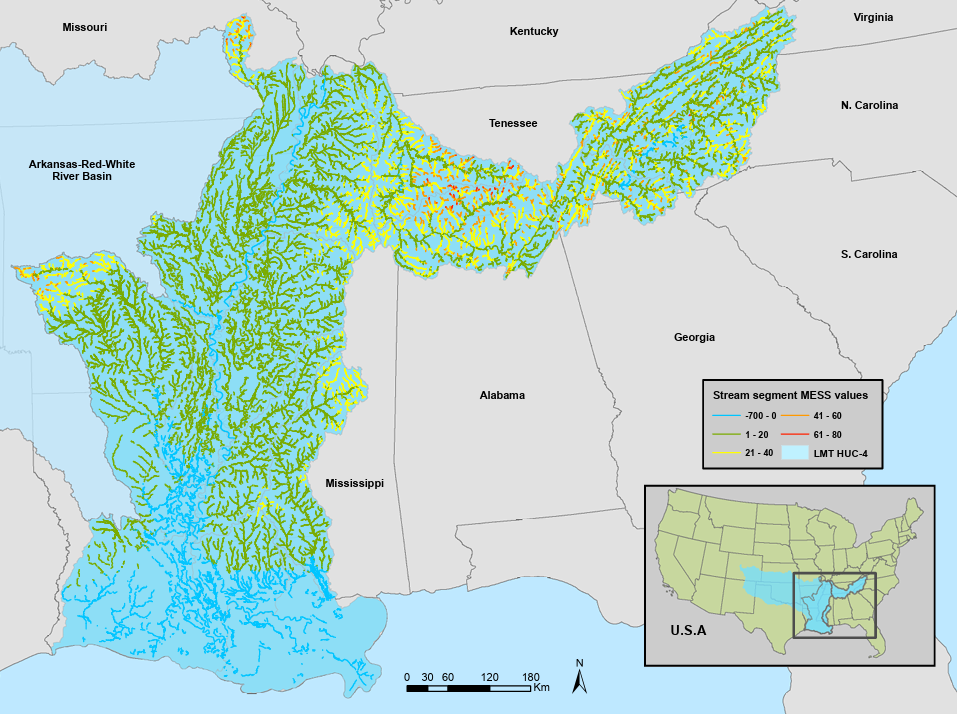


**Figure S4.** Multivariate environmental similarity surfaces (MESS) of the ARW model extrapolated into the LMT range, showing similarity (positive MESS values) and dissimilarity (negative MESS values) of environmental conditions of ARW stream segments in relation to LMT stream segments (stream order ≥3).

**
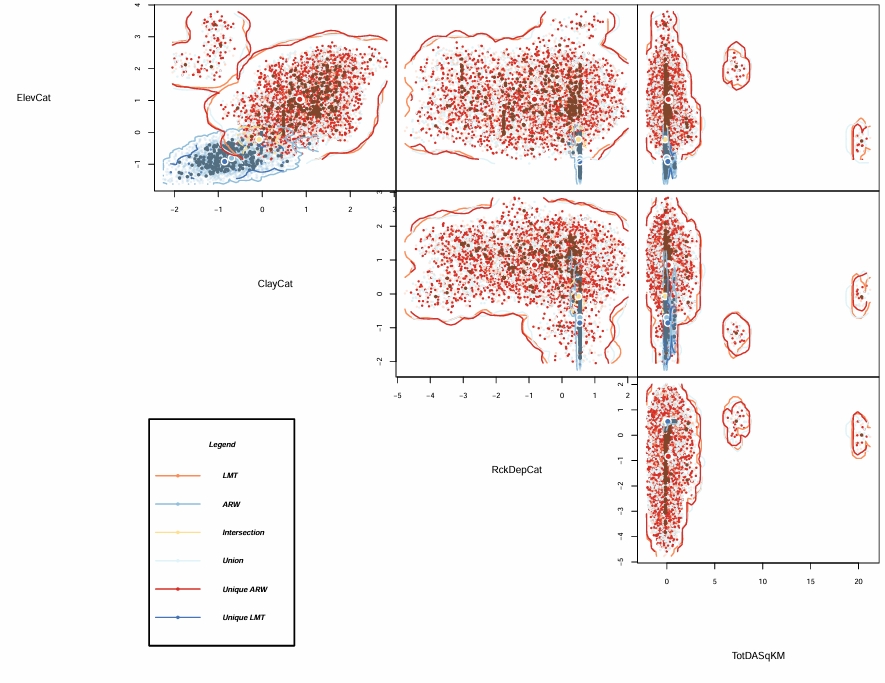
**

**Figure S5.** Visualization of a four-dimensional niche hypervolume of Bluntface Shiner from the Arkansas-Red-White (ARW) and from the Lower Mississippi-Tennessee (LMT).
